# Correlation of shear wave elastography with liver biopsy in children with Chronic Liver disease

**DOI:** 10.12669/pjms.41.2.9886

**Published:** 2025-02

**Authors:** Mehwish Imtiaz, Muhammad Umer Khaqan, Maryam Mazhar, Iqtadar Seerat

**Affiliations:** 1 Mehwish Imtiaz, MBBS, FCPS (Paediatric Medicine) Clinical fellow in Paediatric Gastroenterology and Hepatology, Department of Paediatrics and Child Health, Pakistan Kidney and Liver Institute and Research Centre, Lahore, Pakistan; 2 Muhammad Umer Khaqan, MBBS Medical officer, Department of Paediatrics and Child health, Pakistan Kidney and Liver Institute and Research Centre, Lahore, Pakistan; 3 Maryam Mazhar, MBBS Medical officer, Department of Paediatrics and Child Health, Pakistan Kidney and Liver Institute and Research Centre, Lahore, Pakistan; 4 Iqtadar Seerat, MBBS, MRCPCH (London, UK), FRCPCH (London, UK) Head and Consultant Paediatric Gastroenterology and Hepatology, Pakistan Kidney and Liver Institute and Research Centre, Lahore, Pakistan

**Keywords:** Autoimmune hepatitis, Chronic liver disease, Liver biopsy, Liver fibrosis, Ultrasound shear wave

## Abstract

**Background & Objectives:**

Liver biopsy remains the gold standard for assessment of liver fibrosis; however, it is limited practically because of issues of sampling errors and invasiveness. This opens the way for other important non-invasive processes of diagnosis including the role and utilisation of Shear Wave Elastography in coming years for liver diseases. This post-hoc study will firstly aim to establish the correlation existing between the currently preferred gold standard liver biopsy and 2D-Shear Wave elastography’s diagnostic accuracy in paediatric patients presenting with suspected or confirmed liver disease.

**Methods:**

This retrospective study was conducted in Pakistan Kidney and Liver Institute and Research Center in Lahore, Pakistan from August 2017 to January 2024. In this analysis, 37 Paediatric patients with various liver diseases who underwent liver biopsy for autoimmune hepatitis, Budd-Chiari syndrome, Wilson disease and other liver-related pathologies were included.

**Results:**

Thirty-seven patients with a mean age of 10 years (ranging from 4–14 years) were enrolled. According to this study, sixteen out of thirty seven (43%) cases of chronic liver disease were caused by autoimmune hepatitis. Patients’ average liver stiffness as determined by SWE was 12.14 ± 0.75 kPa. According to elastography, the average liver stiffness in individuals with F0–F1 fibrosis was 6 ± 0.01 kPa, 7.67 ± 0.29 in stage F2, 8.62 ± 0.20 in stage F3, and 14.05 ± 3.69 kPa in stage F4. We discovered that the mean level of hepatic stiffness varied significantly depending on the degree of fibrosis (p = 0.0001).

**Conclusion:**

SWE gauges the liver tissue’s stiffness, which rises with fibrosis severity. Research has demonstrated that SWE, which is frequently equivalent to liver biopsy, has a high degree of accuracy in identifying and staging hepatic fibrosis.

## INTRODUCTION

Chronic liver disease (CLD) is a progressive process of parenchymal destruction and regeneration characterised by fibrosis and potentially culminating in cirrhosis, resulting in impaired liver function. Children with chronic liver disease present a unique set of aetiological factors distinct from adults. Various disorders contribute to CLD in children, encompassing infections, developmental anomalies like biliary atresia (which, if untreated, can progress to biliary cirrhosis), as well as metabolic and neoplastic conditions that can culminate in hepatic failure.^1^ As chronic liver disease advances, the liver parenchyma experiences an increased deposition of fibrous tissue, which eventually results in the development of end-stage cirrhosis.^2^

The severity of fibrosis varies, but there is a clear link between an elevated stage (F3–F4) and a higher chance of clinical problems. These include ascites, hepatic encephalopathy, variceal haemorrhage, portal hypertension, and hepatocellular carcinoma.^2^ There are two stages of cirrhosis: compensated and decompensated. Children who do not exhibit symptoms but have extensive fibrosis or liver cirrhosis are classified as having “compensated advanced chronic liver disease”. Decompensated cirrhosis stands as a significant contributor to both morbidity and mortality. Consequently, assessing the liver’s stage becomes a pivotal element in the management strategy for various liver diseases.^2^ Timely clinical interventions are crucial, as it has the potential to impede the development of end-stage liver disease.

Over the years, LB (liver biopsy) has stood as the most appropriate test for confirming fibrosis and inflammation. However, recent times have seen the approval of several non-intrusive techniques for staging liver fibrosis. This shift is particularly notable due to the associated morbidity and mortality percentages linked with liver biopsy, a risk that escalates with the advanced stages of fibrosis.^2^ A sonographic-based elastography technique called 2-D SWE (2-Dimensional Shear Wave Elastography) is used to quantify liver stiffness non-invasively.

This is accomplished by introducing mechanical waves into human tissue using an ultrasound transducer, the speed of which is correlated with the degree of tissue stiffness. The basic idea is that a softer tissue medium is indicated by 2-D shear wave propagation at lower speeds, and a stiffer tissue medium is indicated by higher speeds. The liver stiffens as fibrosis advances, and 2-D SWE is a useful tool for tracking this change. The main aim of this research was to determine whether LB (liver biopsy), the gold standard for assessing liver fibrosis, and the specificity and sensitivity of SWE are correlated.

## METHODS

Thirty-seven patients were recruited in this retrospective solitary institutional research conducted at Pakistan Kidney and Liver Institute and Research Centre in Lahore, Pakistan. All individuals who had liver biopsies scheduled between August 2017 and January 2024 were included in the study. Every qualified patient received a two-dimensional SWE assessment in the equivalent location as the LB. Every patient had to fast for a minimum of four hours in order to comply with the study protocol. The C1-6-D probe, a convex probe with a frequency range of 1-6 MHz, and the GE-LOGIQ S8 R3.1.9 equivalent software was used to perform shear wave elastography. It was verified that every patient’s BMI was under 34 and that they were fasting for the allotted amount of time in order to assure uniformity.

### Ethical Approval:

It was approved by the ethics committee (PKLI-IRB/AP/180, dated Feb. 13, 2024) and informed consent from each participant.

The patient was laid in a flat position with the right arm overhead for the examination. The intercostal approach was employed, and the Region of Interest (ROI) was placed in the right lobe, specifically in segment VIII, approximately 1-2 cm below Glisson’s capsule, while avoiding major vessels, bile ducts, and any hepatic space-occupying lesions. The shear wave pulse was initiated, ensuring that the ROI box was adequately filled with colour. Measurements were then taken at least 5-10 times in different frames at a single point, with a criterion of at least 60% valid measurements for result calculation.

The aetiology of spectrum for chronic liver disease as mentioned in Table-I. Biopsy was performed by some experienced paediatric hepatologist with the help of 18 G or 16 G automated needle. Two core biopsies were taken from the right lobe of liver. Then the 2-D SWE, was performed. A single, skilled pathologist used the METAVIR scoring system to read the specimens while being blind to the 2-D SWE results. Children with chronic liver diseases and below 15 years were included in the study and children with co-morbidities, acute liver failure and hepatitis B and C infections were excluded. Sensitivity, specificity, positive predictive value & negative predictive value were calculated using SPSS version 26.

**Table-I:** Causes of chronic liver diseases.

	Frequency	Percentage (%)
Autoimmune Hepatitis	16	43.2
Budd Chiari Syndrome	6	16.2
Wilson Disease	5	13.5
Cryptogenic Liver Cirrhosis	4	10.8
Glycogen Storage Disease	2	5.4
Primary Sclerosing Cholangitis	1	2.7
Primary Biliary Cirrhosis	1	2.7
Caroli Disease	1	2.7
Benign recurrent intrahepatic cholestasis (BRIC)	1	2.7

## RESULTS

Thirty-seven patients, ranging in age from 4-14 years, with a mean age of 10, were included in the study. Of them, 40% had various liver illnesses, 16% had Budd-Chiari syndrome, and 43% had autoimmune hepatitis (Table-I).

Table-II provides a full description of the patients’ features. By using elastography, the average liver stiffness was 12.14 ± 0.75 kPa. The mean liver stiffness was 6 ± 0.01 kPa, 7.67 ± 0.29 kPa, 8.62 ± 0.20 kPa, and 14.05 ± 3.69 kPa when stratified by fibrosis stage (F0–F1, F2, F3, and F4). Diagnostic precision of SWE was calculated for finding various fibrosis stages as compared to liver biopsy as shown in Table-III.

**Table-II:** Demographic and clinical features of the patients.

Variable	N=37
Age (mean ± SE)	10±0.515
Aspartate transaminase (AST) (mean ± SE) U/L (normal <34)	113.02±17.14
Alanine transaminase (ALT) (mean ± SE) U/L (normal < 55)	91.02±15.02
Alkaline phosphatase (ALP) (mean ± SE) U/L (normal 35–104)	368.7±38.12
Elastography liver stiffness (kPa) (mean ± SE)	12.29±0.63
INR (mean ± SE)	1.3±0.06
Platelets (mean ± SE) (normal 150-450 10^9/L)	175±21.7

**Table-III:** Diagnostic precision of SWE for finding various fibrosis stages as compared to liver biopsy.

	F0-F1	F2	F3	F4
Sensitivity	0.00 %	10.00 %	10.00 %	81.82 %
Specificity	96.77 %	96.30 %	85.19 %	23.08 %
Positive Predictive Value	0.00 %	49.97 %	19.98 %	31.11 %
Negative Predictive Value	83.35 %	74.31 %	71.90 %	74.94 %

**Fig.1:**
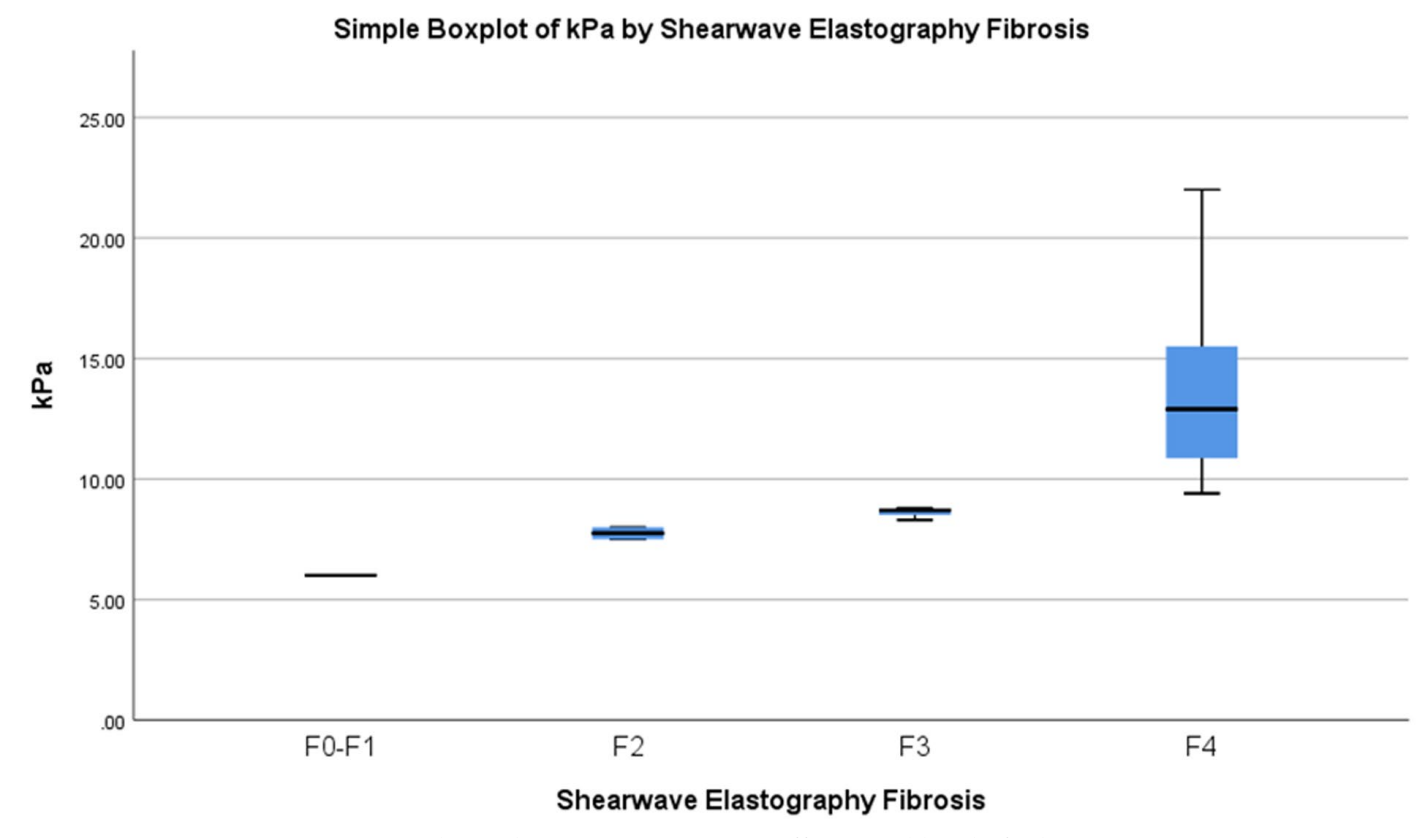
Relation between SWE Liver Stiffness and level of Fibrosis.

Visual representation of liver stiffness measured by elastography categorised by fibrosis stage is depicted in the box and whisker plot. Each box signifies the interquartile range, spanning from the 25th to the 75th percentile, with the median stiffness denoted by a horizontal line within each box. The median stiffness values (in kPa) for different fibrosis stages are as follows: F0–F1: 6.0, F2: 7.67, F3: 8.62, and F4: 14.05, (Fig.1).

The comparison of results of shear wave and liver biopsy showed a significant correlation of results for advanced stage of fibrosis; as 18 among 37 patients’ stage of fibrosis comparable according to both modalities, which comprises 56.8 % (for F3 and F4), (Table-IV).

**Table-IV:** Comparison between elastography fibrosis & Liver biopsy.

		SWE Fibrosis				
		F0-F1	F2	F3	F4	Total
Liver Biopsy (LB) Fibrosis Stage	F0-1	0	1	1	4	6
	F2	1	1	1	7	10
	F3	0	0	1	9	10
	F4	0	0	2	9	11
	Total	1	2	5	29	37

The biomarkers of cirrhosis (ALT and AST), also provides clues for stage of fibrosis as shown in Table-V, the more advanced the stage of fibrosis the decreased the levels of ALT and AST as compared to early stage of fibrosis.

**Table-V:** Comparison of transaminases with different stages of fibrosis.

Liver Fibrosis Stage	Mean	ALT (normal 0-55)	
		SD	p-value
F0-1	91	130.58	0.149
F2	112	113.16	0.012
F3	73.3	54.09	0.002
F4	87.4	81.30	0.005

***Liver Fibrosis Stage***	***Mean***	***AST (normal 5-34)***	
		***SD***	***p-value***

F0-1	140.00	211.92	0.165
F2	114.30	81.00	0.002
F3	103.70	63.32	0.001
F4	105.36	81.22	0.002

## DISCUSSION

The outcome and treatment of CLD are highly dependent on LF. For this reason, the stewardship of such patients, the evaluation of degree of fibrosis plays a vital role.^3^ Despite the fact that LB is still the most reliable investigation for evaluating fibrosis, but it is associated with high-risk side effects because of the invasive nature of test like bleeding and lung complications escalate the requirement of non-invasive tests.^4^,^5^ Recently ultrasound modalities like SWE are reliably used to evaluate the stage of fibrosis. The fundament of SWE is to produce shear waves by tissue displacement.^6^

The purpose of our research was to evaluate and compare the precision of SWE with LB to grade the fibrosis in children of CLD. To the best of our knowledge, this is the first study conducted in this area in Pakistan, where research on this topic is currently limited. The recent search discovered a substantial difference in the mean liver stiffness, as determined by SWE, across patients whose METAVIR scores indicated different stages of hepatic fibrosis. Patients with advanced liver fibrosis showed higher mean liver stiffness than those in the early stages, suggesting that SWE is a useful predictor of varying degrees of liver fibrosis.

Similar findings were reported in studies by Motamed et al^7^ and Cassinotto et al.^8^, where liver stiffness’s mean calculated by SWE was significantly elevated in patients across different intervals of liver fibrosis compared to healthy controls. These studies also demonstrated a notable distinction in liver stiffness between advanced and early fibrosis stages. Guibal et al.’s study^9^ further supported the non-invasive and accurate nature of SWE in assessing liver fibrosis, particularly in diagnosing significant fibrosis across various aetiologies. The research highlighted a direct and meaningful association between liver stiffness assessed by SWE and liver biopsy, employing the METAVIR system. These results lay emphasis on the effectiveness and credibility of SWE as a non-invasive method for assessing liver cirrhosis, providing a reliable method instead of liver biopsy as demonstrated by recent studies.^10^-^12^ SWE results indicate a rising trend in accordance with the advanced stages of fibrosis and has shown to be more beneficial than the blood markers of liver fibrosis. The conclusiveness of SWE in assessing liver fibrosis depends upon the calculation of amount of fibrous tissue by determining the hepatic elastic ration, as indicated by previous studies.^13^

In our study, the sensitivity and specificity for fibrosis stages were 10% and 93.30% (F2), 10% and 85.19% (F3), and 81.82% and 23.08% (F4), respectively. Notably, the highest diagnostic sensitivity was observed for more advanced fibrosis (F4). Meta-analysis results by Li et al.^14^ indicated a high diagnostic accuracy for SWE in F3 (90% sensitivity and 81% specificity) and F4 (87% sensitivity and 88% specificity), with relatively high accuracy in the F2 stage (85% sensitivity and 81% specificity). Guibal et al.^9^ reported sensitivity and specificity of SWE at different stages as 85.1% and 82.7% (F2), 88.9% and 90.3% (F3), 93.3% and 3.98% (F4). Additionally, a significant correlation was found between the percentage of fibrosis and liver stiffness measured by SWE, suggesting a high investigative value for advanced liver fibrosis. Normal and cirrhotic liver generally comprises 5.5mg/g and over 30mg/g of collagen respectively. The histological METAVIR score may not accurately determine the fibrosis. Hence, the accuracy of reporting the stage of fibrosis can be increased by performing LB too.^9^ While liver stiffness serves as a forecaster of liver fibrosis, specific confounding aspects influencing liver stiffness results have been reported. In this regard, certain studies have reported conflicting results.^15^-^17^

Our study revealed a direct and substantial association between liver stiffness at various stages of cirrhosis and AST and ALT readings. The association between transaminases level and stage of cirrhosis. These findings affiliate with the conclusions of the findings by Alempijevic et al.^5^ Kelleher et al. and Verlinden et al. reported an important association between liver stiffness and AST levels.^18^,^19^ Furthermore, other studies have also reported a notable association between AST and ALT levels and liver cirrhosis.^20^,^21^ Other studies in the literature employing SWE revealed no strong link between transaminases level and LB with stiffness.^22^,^23^ The study encompasses a diverse range of liver diseases within the sample population. Despite this diversity, a discernible correlation between fibrosis stage and elasticity readings indicates the likely validity of this method for measuring fibrosis.

### Limitations:

It’s crucial to acknowledge that the majority of our sample exhibits advanced fibrosis, potentially limiting the study’s power in assessing normal/mild fibrosis. Moreover, the distribution of patients among different pathologies results in relatively small numbers in each group. 

## CONCLUSION

Our findings suggest that SWE is a safe and non-invasive technique, exhibiting fewer complications compared to liver biopsy, making it a reliable method to assess the extent of liver cirrhosis/fibrosis in children, irrespective of the underlying cause.

### Author’s Contribution:

**MI:** Conception and design, data analysis and writing original draft of the manuscript. Responsible for accuracy or integrity of the work. **MUK:** Collection of data. Data analysis and interpretation. Manuscript writing, Review and Editing. **MM:** Literature search, Data Collection. **IS:** Concept and design, Data supervision, Critical review. All authors have approved the final version and are accountable for the integrity of the study.
